# Cross-Cultural Adaptation and Validation of the Modern Standard Arabic Versions of the Berlin Questionnaire and Epworth Sleepiness Scale

**DOI:** 10.7759/cureus.82388

**Published:** 2025-04-16

**Authors:** Zainab Lahrichi, Imane El Bassity, Zineb Serhier, Samir Diouny, Adil Khoubila, El Mehdi Jouhadi

**Affiliations:** 1 Department of Fixed Prosthodontics, Faculty of Dental Medicine, Hassan II University of Casablanca, Casablanca, MAR; 2 Clinical Neurosciences and Mental Health Laboratory, Faculty of Medicine and Pharmacy, Hassan II University of Casablanca, Casablanca, MAR; 3 Clinical Neurosciences and Mental Health Laboratory, Faculty of Dental Medicine, Hassan II University of Casablanca, Casablanca, MAR

**Keywords:** berlin questionnaire, cross-cultural adaptation, epworth sleepiness scale, modern standard arabic, morocco, obstructive sleep apnea, validation

## Abstract

Background

The Berlin Questionnaire (BQ) and Epworth Sleepiness Scale (ESS) have been validated in various studies across diverse sociocultural contexts. Despite their significant impact, there is a lack of standardized assessment tools for obstructive sleep apnea syndrome (OSAS) in Arabic-speaking populations. This study aimed to adapt the BQ and the ESS to Modern Standard Arabic (MSA) for use in Moroccan clinical settings and assess their psychometric properties.

Methodology

The BQ and ESS were translated into MSA using a standardized process involving forward and backward translation. The psychometric properties of the Arabic versions of the BQ and ESS were assessed in 125 Moroccan patients recruited from the University Dental Clinic Ibn Rochd in Casablanca at two time points (T1 and T2). Reliability was assessed using Cohen’s kappa index, and internal consistency was measured using Cronbach’s alpha. Associations between variables were analyzed using Pearson’s chi-square and Fisher’s exact tests, with statistical significance set at p-values <0.05.

Results

The BQ identified 50.4% (63 out of 125) of participants as being at high risk for OSAS (95% confidence interval (CI) = 41.8-59.0), while 38.4% (48 out of 125) had a positive ESS score (95% CI = 30.4-47.2). Kappa indices for the BQ ranged from 0.98 to 1.00, while the intraclass correlation coefficient for the ESS was 0.99. Cronbach’s alpha values for the three categories were 0.58, 0.67, and 0.74, respectively. Statistically significant associations were observed between the risk of OSAS and obesity (p = 0.002) and menopause (p = 0.023). Additionally, 61.9% of participants identified as high-risk for OSAS reported experiencing excessive daytime sleepiness (p < 0.001).

Conclusions

The MSA versions of the BQ and ESS demonstrated strong validity and reliability for assessing and diagnosing OSAS in Arabic-speaking populations. These scales represent valuable resources for enhancing clinical practice in settings where Arabic is the primary language.

## Introduction

Obstructive sleep apnea syndrome (OSAS) is a prevalent sleep disorder that affects nearly one billion individuals worldwide [1]. The disorder is characterized by episodes of a complete (apnea) or partial collapse (hypopnea) of the upper airway, accompanied by a decrease in oxygen saturation or arousal from sleep [2]. OSAS is clinically diagnosed based on polysomnographic data, with the Apnea-Hypopnea Index (AHI) serving as a key indicator. For adults, an AHI of more than five events and fewer than 15 events per hour is considered mild OSAS. Moderate OSAS is defined as 15 to 30 events per hour, while severe OSAS is characterized by an AHI of more than 30 events per hour [2]. OSAS affects both physical and mental health [3] and contributes to a range of conditions, including cardiovascular, neurological, endocrine, metabolic, and ophthalmological disorders. Additionally, it causes fragmented sleep and diminished alertness [4]. These effects collectively impair productivity, safety, and overall quality of life.

Polysomnography and respiratory polygraphy are the gold-standard diagnostic tools for sleep apnea [5]. However, for screening, several measures have been developed, including the Berlin Questionnaire (BQ) (Appendix 1) [6,7] and the Epworth Sleepiness Scale (ESS) (Appendix 2) [8], both of which have been validated in several studies across diverse sociocultural settings.

Despite their widespread use, there is a significant lack of validated Arabic versions of these scales for Arabic-speaking adults. This study aimed to fill that gap. The main objectives of the present study were two-fold: (1) to adapt the BQ and the ESS to Modern Standard Arabic (MSA), and (2) to test the psychometric properties of these adapted versions. The results obtained will have implications for early detection and clinical intervention for individuals with OSAS.

## Materials and methods

To address the linguistic and cultural differences between English and Arabic, the BQ and ESS were adapted into MSA following Beaton et al.’s (2000) five-stage process [9]. The first step involved forward translation, where two bilingual translators independently translated the BQ and ESS into MSA. Next, an expert committee reviewed and compared both translations, synthesizing them into a single consensus version. In the back-translation phase, the MSA versions were translated back into English by two independent bilingual translators to ensure consistency with the original versions. The expert committee then carefully reviewed the original and back-translated versions to verify accuracy, cultural relevance, and conceptual equivalence. Finally, the pre-testing phase involved piloting the finalized MSA versions on six patients at the CCTD Ibn Rochd in Casablanca to assess their comprehension of all items and response options. Once the final Arabic versions of the BQ and ESS (Appendices 3 and 4) were produced, the clinical validation phase was initiated through an epidemiological survey conducted over one month, from the beginning to the end of March 2022, at the CCTD Ibn Rochd in Casablanca.

In our study, one interviewer was present to conduct the interviews. This interviewer received comprehensive training to ensure consistency and reliability in data collection. The survey instrument was an anonymous questionnaire consisting of 28 questions aimed at collecting data for the study. It was structured into the following four sections: the first section focused on patient characteristics, the second section screened patients at high and low risk of obstructive sleep apnea (OSA) (BQ), the third section assessed excessive daytime sleepiness (ESS), and the fourth section identified risk factors for OSA.

The BQ consists of 10 questions, along with information on height and weight arranged in the following three categories: (1) snoring and cessation of breathing (five questions), high risk in Category 1 is defined as persistent symptoms in two or more snoring-related questions; (2) excessive daytime sleepiness (four questions), high risk in Category 2 is determined by persistent daytime sleepiness, drowsy driving, or both; and (3) body mass index (BMI) and hypertension (one question), high risk in Category 3 is defined as a history of hypertension or a BMI greater than 30 kg/m². A positive score in two or more categories indicates a high risk for OSA [6].

The ESS consists of eight items representing more or less soporific situations [10,11], namely, describing hypothetical situations such as sitting and reading, watching television, sitting inactive in a public space, as a passenger in a car for an hour without a break, lying down to rest in the afternoon when circumstances permit, sitting and talking to someone, sitting quietly after a lunch without alcohol, and in a car while stopped for a few minutes in traffic [8,12]. Scores range from 0 to 3 (0 = none; 1 = slight; 2 = moderate; 3 = high), resulting in a total score ranging from 0 to 24 points [8,12]. According to Johns [8], a higher score suggests a higher propensity to fall asleep. Conventionally, scores above or equal to 10 may indicate sleep disorders.

The study included 125 patients attending the CCTD Ibn Rochd in Casablanca who agreed to voluntarily answer the questionnaire. Participants had to be over 30 years of age and have attained at least a primary school education. Patients previously diagnosed with OSA, those with facial trauma, and those with bruxism were excluded from the study. Pregnant women, patients with occlusion disorders, and those working at night were also excluded. The study’s objective was explained to the patients before they completed the questionnaire, following which they provided verbal consent. After completion, the BQ and ESS scores were calculated in the patient’s presence. Patients completed the questionnaire once (T1) and a second time (T2), either an hour later for some or a week later for those with a follow-up appointment.

The psychometric properties of the final MSA versions of the BQ and ESS were evaluated based on reliability (internal consistency and test-retest reliability) and validity. Internal consistency was measured using Cronbach’s alpha, with a threshold of 0.6 set to determine scale reliability. Test-retest reliability was assessed using the kappa index and the intraclass correlation coefficient (ICC) to evaluate consistency over time, with all participants completing the questionnaires twice. Statistical analysis was conducted using a Student’s t-test and chi-square test to explore the association between variables and verify construct validity. A p-value ≤0.05 was considered statistically significant. Data were entered into Microsoft Excel version 2021 (Microsoft Corp., Redmond, WA, USA) and analyzed using SPSS version 25.0 (IBM Corp., Armonk, NY, USA).

## Results

The study initially included 135 patients, resulting in a response rate of 92.6% (125 completed questionnaires returned). Demographic information of the participants is shown in Table 1. Of the participants, 57.6% were female, the mean age was 47.7 years (SD = 11.2), 39.2% had a university level of education, and 24% had a secondary school level of education. Additionally, 9.6% of patients were smokers, 4.8% consumed alcohol, 27.2% had nasal obstruction, and 35.6% of female participants had gone through menopause.

**Table 1:** Sociodemographic data and frequency of risk factors of sleep apnea of the study sample. BMI: body mass index

		Number	Percentage (%)
Gender	Female	72	57.6
	Male	53	42.4
Age, mean (SD) = 47.7 (11.2)	<45 years old	51	40.8
	≥45 years old	74	59.2
BMI, mean (SD) = 26.7 (4.6)	≤30	101	80.8
	>30	24	19.2
Level of education	Primary	30	24.0
	Lower secondary	16	12.8
	Upper secondary	30	24.0
	University	49	39.2
Alcohol		6	4.80
Smoking		12	9.6
Nasal obstruction		34	27.2
Menopause		26	36

Table 2 highlights the number and proportion of participants at high risk of OSAS according to the BQ, as well as those experiencing excessive daytime sleepiness based on the ESS. According to the BQ, 50.4% of patients were identified as being at high risk of OSAS, with a 95% confidence interval (CI) of 41.8%-59.0%. Additionally, 38.4% of patients had a positive ESS, with a 95% CI of 30.4%-47.2%.

**Table 2:** Frequency of patients at high risk of apnea according to the BQ and high risk of daytime sleepiness according to the ESS. *: A positive score in two or more categories of the questionnaire indicates a high risk. **: Scores above or equal to 10 indicate the presence of sleep disorders. CI: confidence interval; OSAS: obstructive sleep apnea syndrome; BQ: Berlin Questionnaire; ESS: Epworth Sleepiness Scale

	Number (n = 125)	Percentage (%)	95% CI
OSAS according to the BQ*	63	50.4	41.8-59.0
Sleepiness according to the ESS**	48	38.4	30.4-47.2

Table 3 presents data about the reliability of the BQ and the ESS. The kappa index for the first category of the BQ was 0.98, indicating almost perfect agreement. For the second and third categories of the BQ, the kappa index was 1.00, indicating perfect agreement. For the ESS, the ICC was 0.99, indicating almost perfect agreement. The internal consistency of the questionnaire was assessed using Cronbach’s alpha. The values obtained were 0.58 for the first category of the BQ, 0.67 for the second category, and 0.74 for the ESS, indicating acceptable internal reliability.

**Table 3:** Internal consistency (reliability) and reproducibility of BQ and ESS. The first category of BQ is snoring and cessation of breathing. The second category of BQ is excessive daytime sleepiness. The third category of BQ is BMI and hypertension. *: ICC. BQ: Berlin Questionnaire; ESS: Epworth Sleepiness Scale; ICC: intraclass correlation coefficient; BMI: body mass index

	Number of questions	Kappa index/ICC	Cronbach’s alpha index
First category of BQ	5	0.988	0.58
Second category of BQ	4	1.00	0.67
Third category of BQ	1	1.00	-
ESS	-	0.99*	0.74

Discriminant validity was evaluated by analyzing associations between variables, as shown in Table 4. The association between the risk of apnea (positive BQ) and sociodemographic characteristics and risk factors for sleep apnea was statistically significant for obesity (p = 0.002) and menopause (p = 0.023) . Obese people were at higher risk of OSAS (79% vs. 44%), and the frequency of OSAS was higher among menopausal women (69% vs. 41%). The associations between excessive daytime sleepiness according to the ESS and all sociodemographic characteristics and risk factors for sleep apnea were not statistically significant.

**Table 4:** Association between the risk of sleep apnea syndrome and excessive daytime sleepiness with sample characteristics and sleep apnea risk factors. P-value: probability value; χ²: chi-square test; *: Fisher’s exact test was used when some expected frequencies were less than 5.

		Berlin positive			Epworth score ≥10		
		Number (%)	P-value	χ²	Number (%)	P-value	χ²
Gender	Female	37 (51.4)	0.797	0.067	29 (40.4)	0.615	0.254
	Male	26 (49.1)			19 (35.9)		
Obesity	Obese	19 (79.2)	0.002	9.833	13 (54.2)	0.077	3.122
	Not obese	44 (43.6)			35 (34.7)		
Smoking	Smokers	6 (50.0)	0.977	0.001	6 (50.0)	0.578	0.311
	Non-smokers	57 (50.5)			42 (37.2)		
Alcohol	Alcoholics	3 (50.0)	1	--*	45 (37.9)	0.675	--*
	Non-alcoholics	60 (50.5)			3 (50.0)		
Nasal obstruction	Yes	22 (64.8)	0.051	3.824	14 (41.2)	0.696	0.153
	No	41 (45.1)			34 (37.4)		
Menopause	Yes	18 (69.3)	0.023	5.186	12 (46.2)	0.445	0.585
	No	19 (41.3)			17(37.0)		

Table 5 shows the associations between high risk of OSAS according to the BQ and the excessive daytime sleepiness according to ESS. The p-value was <0.001, indicating that this association is statistically significant. Among the patients at high risk of OSAS according to the BQ, 61.9% reported excessive daytime sleepiness according to the ESS. These results suggest that the questionnaire may be considered reasonably valid and reliable.

**Table 5:** Associations between high risk of OSAS according to the BQ and the excessive daytime sleepiness according to the ESS. P-value: probability value; χ²: chi-square. OSAS: obstructive sleep apnea syndrome; BQ: Berlin Questionnaire; ESS: Epworth Sleepiness Scale

		Epworth Score ≥10		P-value	χ²
		Number	Percentage		
Berlin score	Positive	39	61.9	<0.001	29.67
	Negative	9	14.5		

## Discussion

Given the significant prevalence of OSAS and its well-documented impact on both physical and mental health, systematic screening plays a critical role in the early identification and management of this disorder [13]. Therefore, it is crucial to validate simple tools for early diagnosis. Our work on the translation and cross-cultural adaptation of the BQ and ESS is particularly significant for one main reason, i.e., the relevance of these tools in diagnosing and managing patients with suspected apnea/hypopnea. The choice of MSA as the target language for these translations was driven by the objective of extending the use of these tools across North Africa. This ensures broader comprehension in neighboring countries where the Moroccan dialect may not be widely understood.

The internal consistency of the BQ and the ESS was measured using Cronbach’s alpha, a psychometric statistic that assesses the reliability of questionnaire items, with values closer to 1.0 indicating higher reliability. Generally, an alpha of 0.7 is considered acceptable [14]. Cronbach’s alpha coefficient was 0.58 for the first category of the BQ, 0.67 for the second category, and 0.74 for the ESS, indicating acceptable internal consistency in the latter two categories in both instruments. Although the alpha value of 0.58 is slightly below the commonly accepted threshold of 0.7, it is relatively close and may still be considered acceptable.

These results are satisfactory but somewhat lower than those reported in validation studies by Sharma et al. and Netzer et al. [6,15], who reported Cronbach’s alpha values of 0.92-0.96 and 0.86-0.92, respectively. Similarly, a Thai validation study of the BQ reported an acceptable Cronbach’s alpha of 0.68 [16]. However, another Thai study on the ESS achieved a higher Cronbach’s alpha of 0.87, and the Japanese version of the ESS demonstrated good reliability, with a Cronbach’s alpha of 0.83 [17].

The reproducibility of the BQ, assessed through test-retest reliability for categories 1, 2, and 3, showed almost perfect agreement with kappa values of 0.98, 1.00, and 1.00, respectively. This indicates nearly perfect ICC. The Thai version also showed high reliability with a kappa value of 0.97 [16], consistent with the Korean validation [18], which reported an almost perfect kappa of 0.92.

In our sample, statistically significant associations were observed between risk of apnea (BQ) and obesity (p = 0.002), with 79.2% at high risk of OSAS, and risk of apnea (BQ) and menopause (p = 0.023), with 69.3% at high risk of OSAS. These findings align with existing research indicating that obesity and menopause are predisposing factors for sleep apnea [19]. Conversely, associations between the risk of apnea (BQ) and gender, smoking, alcohol use, and nasal obstruction were not statistically significant, with p-values of 0.797, 0.977, 0.100, and 0.051, respectively. In the present study, among patients with high risk of OSAS according to the BQ, 61.9% also reported excessive daytime sleepiness according to the ESS.

During this survey, we encountered several difficulties. Some patients refused to complete the questionnaire, while others were unwilling to wait to fill it out a second time. The intervals between assessments varied based on participant availability, with some participants reassessed an hour later and others a week after their initial appointment. This variability could potentially impact the test-retest reliability of our measurements. However, previous research comparing two-day and two-week intervals found no significant differences in reliability for health status instruments. Nonetheless, we acknowledge this variability as a limitation and recommend that future studies explore the impact of such time interval differences on measurement reliability. Additionally, some participants returned incomplete questionnaires, leaving certain sections blank. Nevertheless, it is important to emphasize that the majority of respondents showed a genuine interest in the subject, particularly those experiencing symptoms of sleep apnea. However, overall, our study yielded a reliable and valid version of these questionnaires suitable for clinical use.

## Conclusions

The BQ and ESS are valuable tools for assessing daytime sleepiness, screening for OSAS, and aiding dentists in managing sleep disorders, particularly sleep apnea syndrome. The Arabic translation of these tools has demonstrated sufficient internal consistency and reproducibility in Moroccan patients, making them reliable resources for screening and prevalence studies of OSAS risk among literate Arabic-speaking populations.

## Figures and Tables

**Figure 1:**
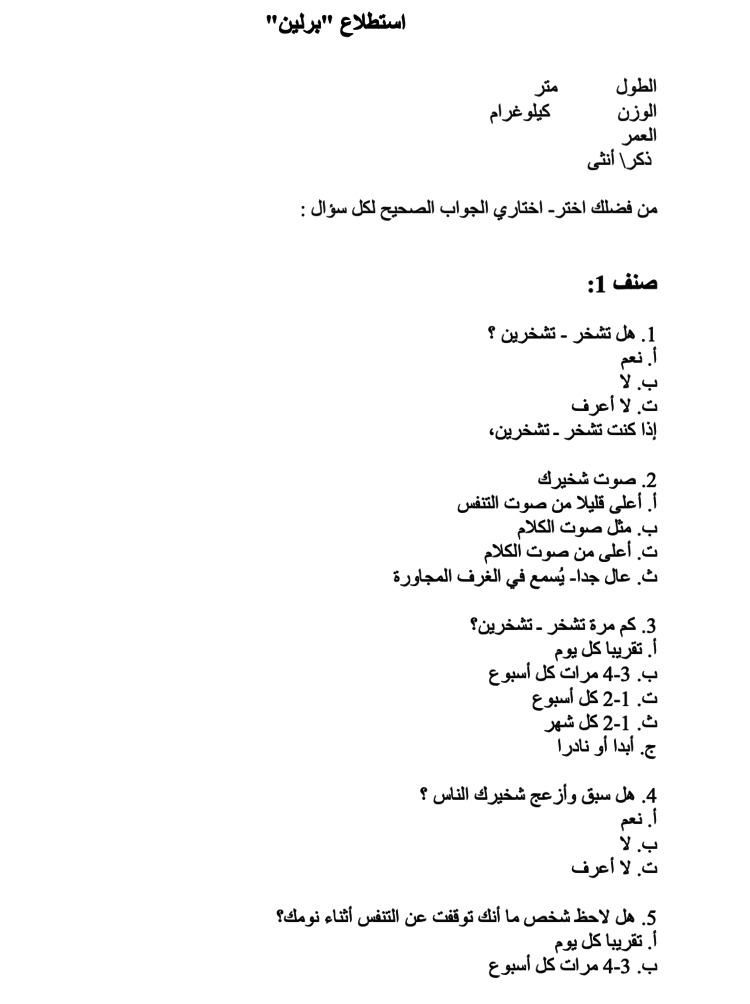
The first part of the Arabic version of the Berlin Questionnaire.

**Figure 2:**
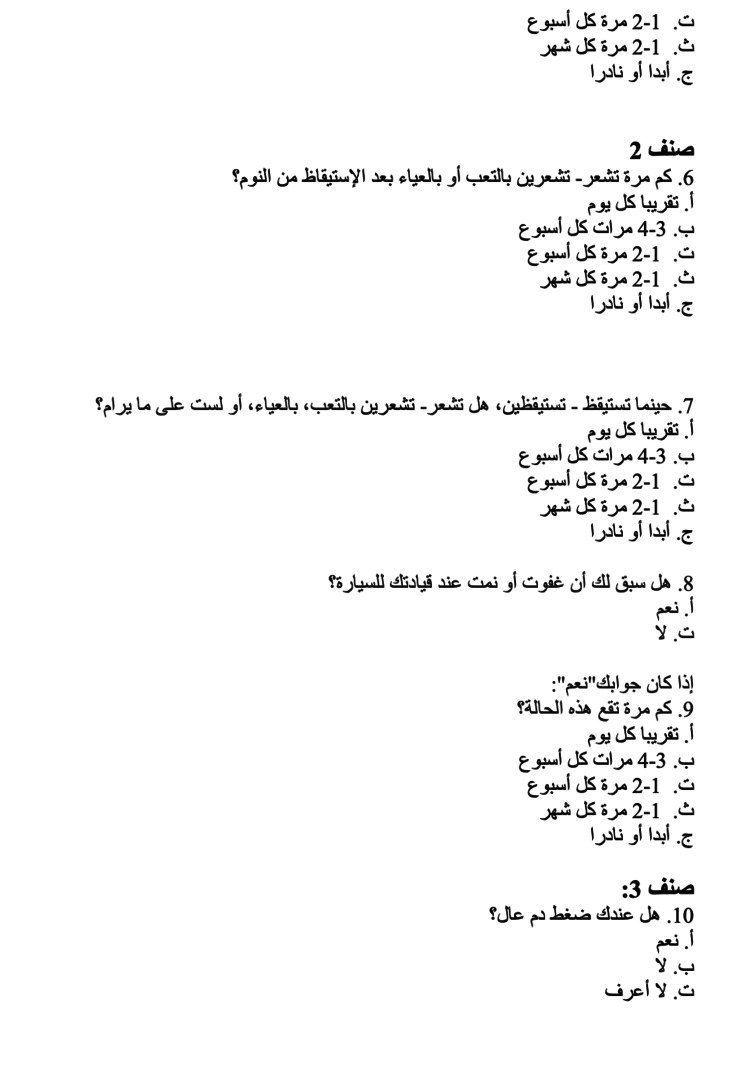
The second part of the Arabic version of the Berlin questionnaire.

**Figure 3:**
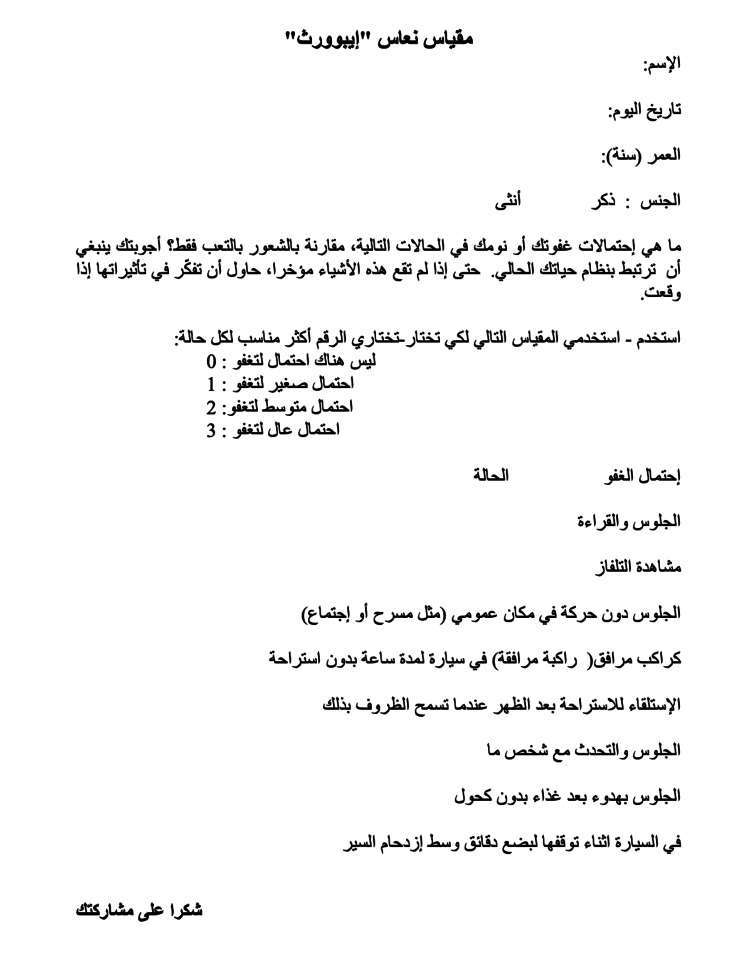
The Arabic version of the Epworth Sleepiness Scale.

**Figure 4:**
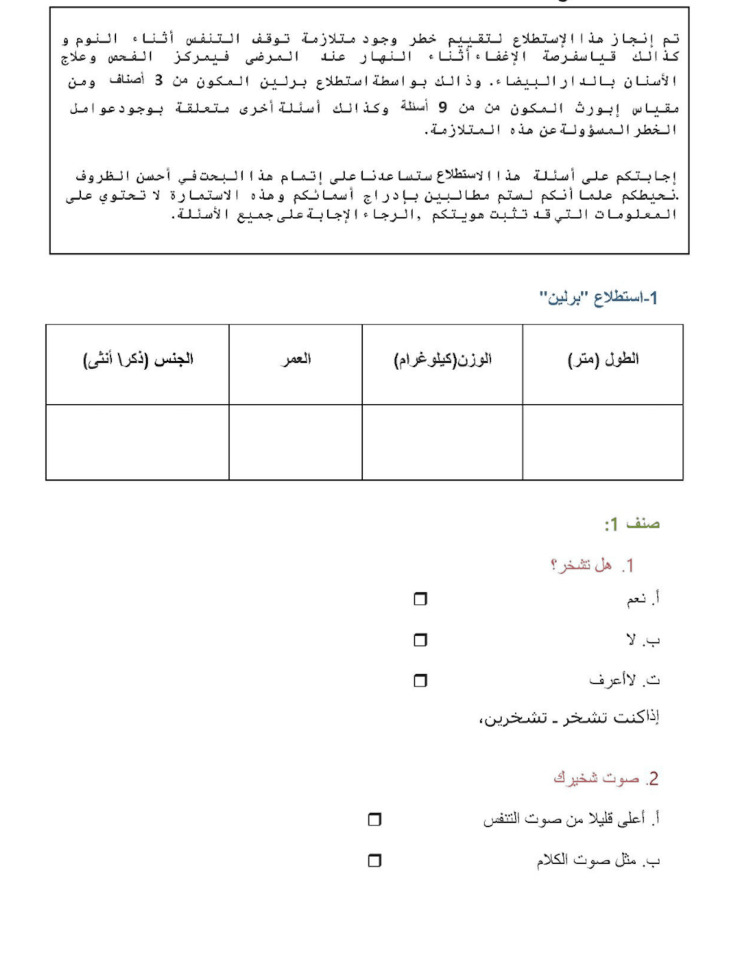
The survey questionnaire (page 1).

**Figure 5:**
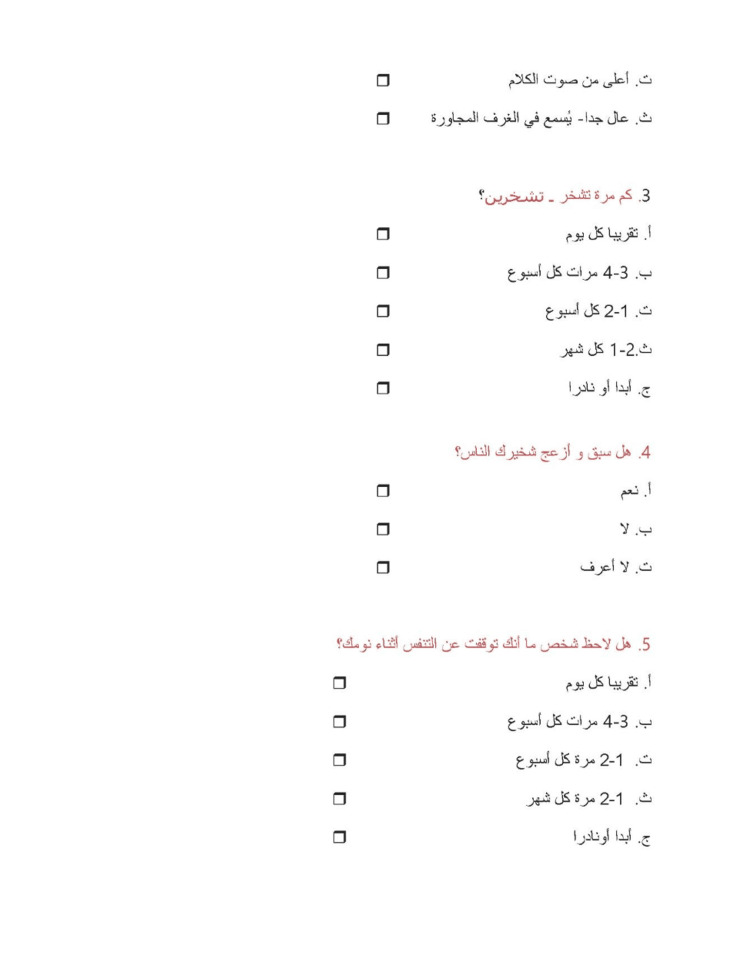
The survey questionnaire (page 2).

**Figure 6:**
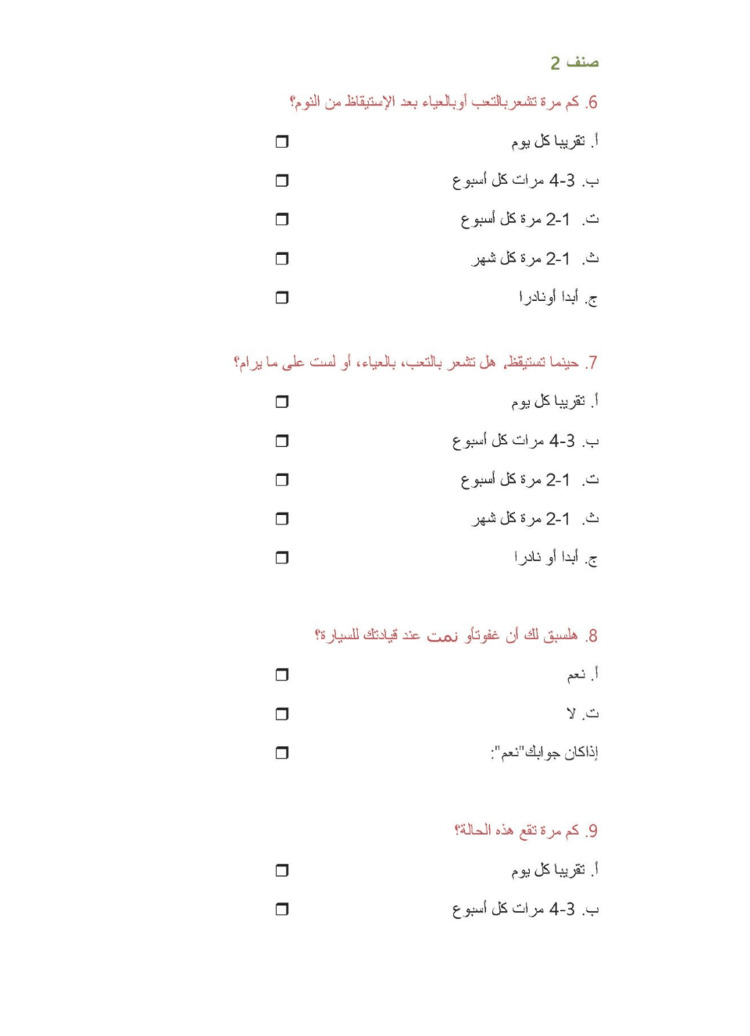
The survey questionnaire (page 3).

**Figure 7:**
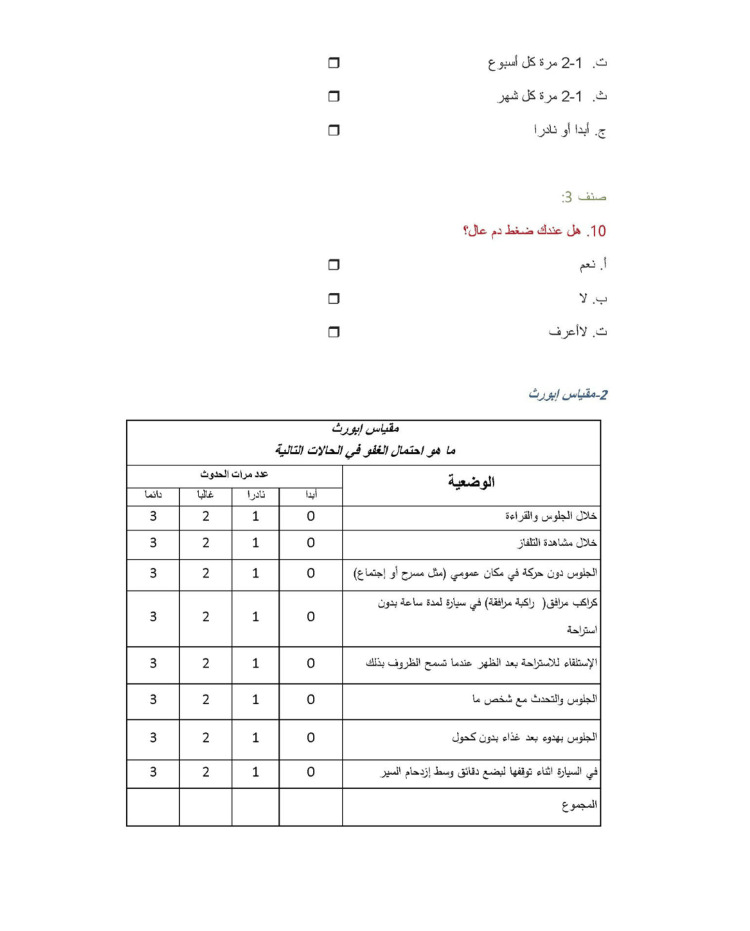
The survey questionnaire (page 4).

**Figure 8:**
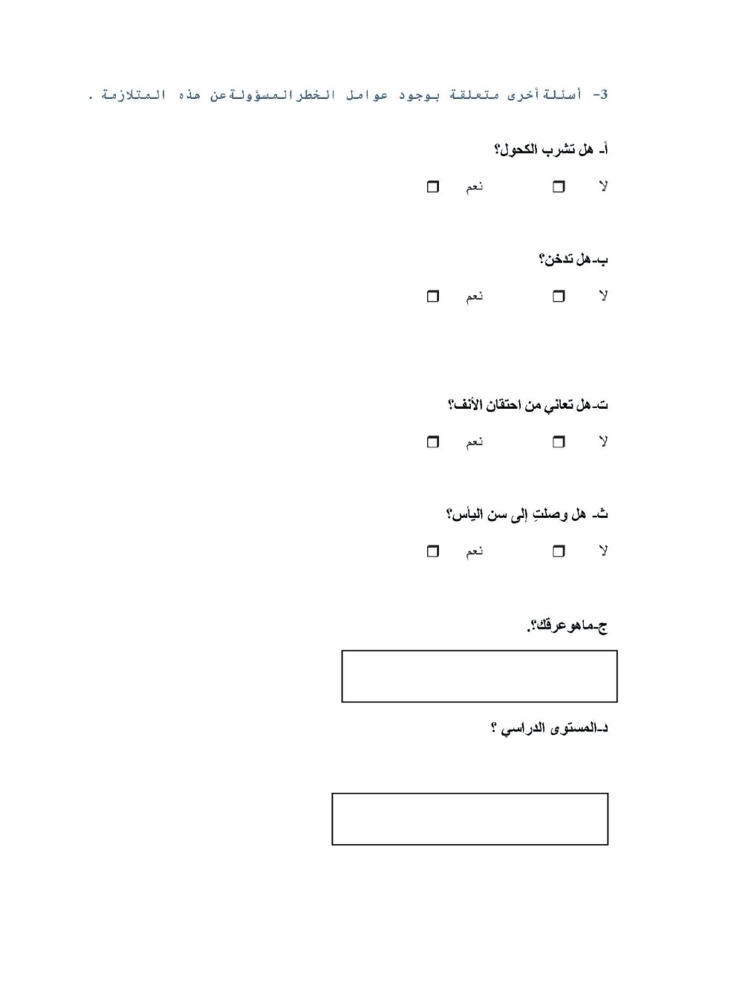
The survey questionnaire (page 5).

## Appendices

Appendix 1: Berlin Questionnaire

Height _____ m Weight _____ kg Age_____ Male/Female

Please choose the correct response to each question.

Category 1

1. Do you snore?

a. Yes

b. No

c. Don’t know

If you snore:

2. Your snoring is:

a. Slightly louder than breathing

b. As loud as talking

c. Louder than talking

d. Very loud-can be heard in adjacent rooms

3. How often do you snore?

a. Nearly every day

b. 3-4 times a week

c. 1-2 times a week

d. 1-2 times a month

e. Never or nearly never

4. Has your snoring ever bothered other people?

a. Yes

b. No

c. Don’t know

5. Has anyone noticed that you quit breathing during your sleep?

a. Nearly every day

b. 3-4 times a week

c. 1-2 times a week

d. 1-2 times a month

e. Never or nearly never

Category 2

6. How often do you feel tired or fatigued after your sleep?

a. Nearly every day

b. 3-4 times a week

c. 1-2 times a week

d. 1-2 times a month

e. Never or nearly never

7. During your waking time, do you feel tired, fatigued, or not up to par?

a. Nearly every day

b. 3-4 times a week

c. 1-2 times a week

d. 1-2 times a month

e. Never or nearly never

8. Have you ever nodded off or fallen asleep while driving a vehicle?

a. Yes

b. No

If yes:

9. How often does this occur?

a. Nearly every day

b. 3-4 times a week

c. 1-2 times a week

d. 1-2 times a month

e. Never or nearly never

Category 3

10. Do you have high blood pressure?

a. Yes

b. No

c. Don’t know

Appendix 2: The Epworth Sleepiness Scale

Name:

Today’s date:

Your age (years):

Your sex (male = M; female = F):

How likely are you to doze off or fall asleep in the following situations, in contrast to feeling just tired? This refers to your usual way of life in recent times. Even if you have not done some of these things recently, try to work out how they would have affected you.

Use the following scale to choose the most appropriate number for each situation:

0: Would never doze

1: slight chance of dozing

2: moderate chance of dozing

3: high chance of dozing

Situation                           Chance of dozing

Sitting and reading          ………….

Watching TV                  ………….

Sitting, inactive in a public place

(e.g., a theater or a meeting)      ………….

As a passenger in a car for an hour without

a break                                       ………….

Lying down to rest in the afternoon when

circumstances permit                 ………….

Sitting and talking to someone ………….

Sitting quietly after a lunch without 

alcohol                                        ………….                                                                            

In a car, while stopped for a few minutes

in the traffic                                ………….

Thank you for your cooperation.

Appendix 3: Arabic version of the Berlin Questionnaire

Appendix 4: The Arabic version of the Epworth Sleepiness Scale

Appendix 5: Survey questionnaire
